# Gut Microbial Signatures of Distinct Trimethylamine N-Oxide Response to Raspberry Consumption

**DOI:** 10.3390/nu14081656

**Published:** 2022-04-15

**Authors:** Maximilien Franck, Juan de Toro-Martín, Thibault V. Varin, Véronique Garneau, Geneviève Pilon, Denis Roy, Patrick Couture, Charles Couillard, André Marette, Marie-Claude Vohl

**Affiliations:** 1 Institute of Nutrition and Functional Foods (INAF), Université Laval, Quebec, QC G1V 0A6, Canada; maximilien.franck.1@ulaval.ca (M.F.); juan.de-toro-martin.1@ulaval.ca (J.d.T.-M.); thibaut.varin.1@ulaval.ca (T.V.V.); veronique.garneau@fsaa.ulaval.ca (V.G.); genevieve.pilon@criucpq.ulaval.ca (G.P.); denis.roy@fsaa.ulaval.ca (D.R.); patrick.couture@fmed.ulaval.ca (P.C.); charles.couillard@fsaa.ulaval.ca (C.C.); andre.marette@criucpq.ulaval.ca (A.M.); 2 Centre Nutrition, Santé et Société (NUTRISS), Université Laval, Quebec, QC G1V 0A6, Canada; 3 School of Nutrition, Université Laval, Quebec, QC G1V 0A6, Canada; 4 Quebec Heart and Lung Institute (IUCPQ) Research Center, 2725 Chemin Sainte-Foy, Quebec, QC G1V 4G5, Canada; 5 Endocrinology and Nephrology Unit, CHU de Quebec Research Center, Quebec, QC G1V 4G2, Canada

**Keywords:** raspberry, gut microbiota, TMAO, metabolic disorders

## Abstract

The aim of this exploratory study was to evaluate the gut microbial signatures of distinct trimethylamine N-oxide (TMAO) responses following raspberry consumption. Investigations were carried out in 24 subjects at risk of developing metabolic syndrome who received 280 g/day of frozen raspberries for 8 weeks. Blood and stool samples were collected at weeks 0 and 8. Inter-individual variability in plasma TMAO levels was analyzed, 7 subjects were excluded due to noninformative signals and 17 subjects were kept for analysis and further stratified according to their TMAO response. Whole-metagenome shotgun sequencing analysis was used to determine the impact of raspberry consumption on gut microbial composition. Before the intervention, the relative abundance of Actinobacteriota was significantly higher in participants whose TMAO levels increased after the intervention (*p* = 0.03). The delta TMAO (absolute differences of baseline and week 8 levels) was positively associated with the abundance of gut bacteria such as *Bilophila wadsworthia* (*p* = 0.02; r^2^ = 0.37), from the genus *Granulicatella* (*p* = 0.03; r^2^ = 0.48) or the *Erysipelotrichia* class (*p* = 0.03; r^2^ = 0.45). Changes in the gut microbial ecology induced by raspberry consumption over an 8-week period presumably impacted quaternary amines-utilizing activity and thus plasma TMAO levels.

## 1. Introduction

In the last decade, the studies of gut microbiota shifted to more functional approaches, such as metabolism, inter-species interactions, the impact of the host environment on the microbial ecosystem functioning and the related health effects. Accordingly, the development of high-throughput DNA sequencing and omics technologies allowed for a deeper understanding of the host–microbiota dialog by facilitating the establishment of links among diet, modulation of the microbial ecosystem and health effects on the host, in particular by defining the role of the metabolism of intestinal microbes in the trajectory of biomolecules. The generation of trimethylamine N-oxide (TMAO), a prototypic metabolite of the gut–liver axis, provides a good illustration of this paradigm. It is derived from dietary choline, betaine and L-carnitine, which are hydrolyzed by gut microbes to generate trimethylamine (TMA), which in turn is oxidized by hepatic-flavin-containing monooxygenases to produce TMAO [1]. Particular attention has been drawn to its origin and metabolism due to its proinflammatory and proatherogenic activities and its association with metabolic dysfunction [2,3].

We previously investigated the immune-metabolic changes in response to raspberry (Rb) consumption and found that 10 plasma metabolites were significantly altered, among which an increase in plasma TMAO concentrations was noted after Rb consumption [4]. Nonetheless, the possible link between changes in plasma TMAO and gut microbiota was not explored. In the present study, we hypothesized that the inter-individual variability in plasma TMAO levels in response to Rb consumption is attributable to a pre-existing heterogeneity in gut microbiota profiles. To verify this hypothesis, subjects were stratified into two groups according to their plasma TMAO levels throughout the intervention. Subsequently, fecal microbial profiles and metabolic syndrome (MetS)-related phenotypes were compared. The aim was to highlight potential links between specific gut microbiota signatures and the heterogeneous TMAO response to Rb. The stratification of study participants on the basis of TMAO responses, and the comparison of their fecal microbiota makeup, led to the identification of distinctions at several taxonomic ranks from phyla to species, prior to the intervention. Certain of these bacterial species were correlated with plasma TMAO levels, consistent with the potential contribution of microbiota-related mechanisms to divergent plasma TMAO responses following Rb consumption.

## 2. Materials and Methods

### 2.1. The Study Design and Data Collection

Data used in the present study stem from a previous clinical trial, which aimed to explore the effects of Rb consumption during 8 weeks on MetS risk factors, as well as in the blood transcriptomic and metabolomic profiles and in the composition of gut microbiota in subjects with an altered metabolic profile [4]. The clinical trial was approved by the Université Laval Ethics Committee and registered at clinicaltrials.gov (accessed on 2 February 2022) (NCT03620617). All participants signed an informed consent form. The protocol is described in detail elsewhere [4]. Participants of the clinical trial were Caucasian men or pre-menopausal women between 18 and 60 years old, with a body mass index (BMI) between 25 and 40 kg/m^2^ or a waist circumference ≥80 cm for women or ≥94 cm for men. Inclusion criteria also included plasma triglycerides >1.35 mmol/L or insulin concentration > 42pmol/L [5]. Thus, a total of 59 participants were randomly assigned to maintain their usual diet (*n* = 30) or to consume 280 g/day of frozen Rb (*n* = 29). From the initial 59 participants, 11 withdrew during follow-up, 5 from the Rb-treated group and 6 from the control group. Physical examination, food frequency questionnaires and biological sample collection were conducted before and after the Rb consumption, at weeks 0 and 8, respectively [4]. Blood pressure as well as blood levels of insulin, glucose and lipids were measured with standard protocols, as described previously [4]. Plasma lipopolysaccharide (LPS) and LPS-binding protein levels were measured by using ELISA Kits from Cusabio Technology LLC (Houston, TX, USA) and R&D Systems Inc. (Minneapolis, MN, USA), respectively. In the present study, only the data from the 24 participants assigned to the Rb-treated group were used.

### 2.2. TMAO Profiling and Patient Stratification

Plasma samples were collected before and after the Rb consumption, at weeks 0 and 8, and subsequently analyzed at the Analytical Facility for Bioactive Molecules (Hospital for Sick Children, Toronto, ON, Canada) with an MxP^®^ Quant 500 kit for targeted metabolic profiling (Biocrates Life Sciences AG, Innsbruck, Austria), as previously described [4]. A combination of flow injection analysis (FIA) and liquid-chromatography-based triple quadrupole mass spectrometry (LC-MS/MS) was used to analyze plasma TMAO levels. The MetaboAnalystR package (v3.0) [6] was used for TMAO raw data preprocessing, including the filtering of noninformative signals based on the interquartile range. A total of 17 participants from the Rb-treated group had TMAO measurements that passed quality filters. The retained TMAO measurements were then normalized (quantile normalization), transformed (logarithmic transformation) and scaled (Pareto scaling). Finally, we identified TMAO subgroups based on the change in TMAO values following the consumption of Rb.

### 2.3. Fecal Metagenomics

Stool samples were collected before and after the Rb consumption, at weeks 0 and 8, and stored at −80 °C for further analysis. Fecal microbiota composition from the participants was analyzed. The method of bacterial DNA extraction is described in detail elsewhere [7]. Sequencing was conducted at the Genome Quebec Innovation Centre. Illumina HiSeq 2500 technology (San Diego, CA, USA) was used for whole-genome sequencing of stool samples. An average of 200 million 125 bp paired-end reads per lane were generated. Trimming and quality control were performed on paired reads using Trimmomatic (v0.40) with a cutoff ≥ Q20 [8]. Bowtie2 (v2.4.2) [9] was used for mapping reads to the human hg38 genome. SLIMM (v0.3.0) [10] was used to annotate taxonomically host-decontaminated reads at the species level. Species that showed an abundance <0.1% and were not observed in more than three participants were removed from further analyses. Identification of differentially abundant species between groups and timepoints was performed with deseq2 (v1.34.0) [11]. Results are expressed as log2 fold change (FC) of abundance, and *p*-values were adjusted for multiple testing using false discovery rate (FDR). Phylum relative abundance was estimated by adding the relative abundance of taxa that belong to the same phylum and then normalizing them to 100. Data were rarefied before calculating alpha diversity analysis to adjust for differences in library sizes across samples. Alpha diversity was analyzed by using the Shannon index and the Simpson’s reciprocal index. HUMAnN 2.0 [12] was used to generate pathway abundance and pathway coverage for each sample. Only functional pathways with a coverage score >0.1 in at least 50% of samples were kept for downstream analyses. The relative abundance of functional pathways was estimated by adding the relative abundance of species associated with the same functional pathway and then normalizing this to 100. Only functional pathways showing a relative abundance greater than 10% were kept.

### 2.4. Statistical Analyses

Skewness and kurtosis were used to assess the normality of clinical and metabolic variables. Mean and standard deviation were used to present descriptive characteristics of variables. We assessed differences in baseline levels and in changes throughout the study by using χ^2^ tests for categorical variables and the analysis of variance for continuous variables (general linear models, type III sum of squares). Differences in clinical variables between groups were assessed with the use of the MIXED procedure for repeated measures in the SAS version 3.8 (SAS Institute, Cary, NC, USA) to examine the effects of groups, time and their interaction. Analyses were adjusted for BMI, sex and age, as well as for baseline levels or study variables. Wilcoxon rank sum test was used to analyze differences in alpha diversity. The association between the abundance of bacterial species and delta values of both plasma TMAO and LBP levels was tested by linear regression. An unadjusted mixed linear model was performed to compare groups throughout the intervention using nlme package. A result was considered statistically significant when *p*-value < 0.05. Multiple-testing correction was not applied to the present results. R v4.0.5 was used to perform all metagenomic analyses [13].

## 3. Results

### 3.1. Inter-Individual Variability in Plasma TMAO Levels in Pre- versus Post-Rb Consumption

**Figure 1:**
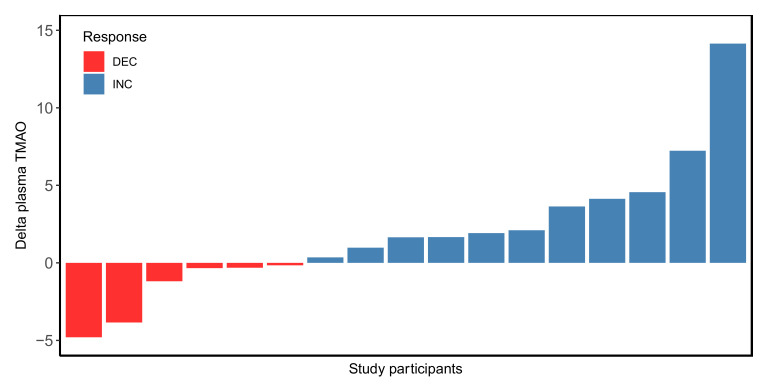
Individual plasma changes in TMAO levels after 8-week raspberry consumption. The *y* axis represents delta values in plasma TMAO in pre- versus post-Rb consumption. Each bar on the *x* axis represents one participant. DEC (red bars) and INC (blue bars) stand for TMAO decrease and TMAO increase subgroups, respectively.

Plasma TMAO levels globally increased in response to the intervention in the Rb-treated group, with a mean increase of 1.86 ± 4.3 μmol/L (Figure S1A), as we previously reported [4]. This result was significantly different to the plasma TMAO change observed in the control group (*p* = 0.01), which showed a mean decrease of –1.36 ± 2.9 μmol/L (*n* = 21) (Figure S1A). Further analysis of this result revealed that while a nonsignificant variation in plasma TMAO levels was observed in the control group between weeks 0 and 8 (4.52 ± 0.61 vs. 3.16 ± 0.68; *p* = 0.1), a significant increase was observed in the Rb group (3.91 ± 0.67 vs. 5.78 ± 0.75; *p* = 0.04) (Figure S1B). These results also reveal a large inter-individual variability in the plasma TMAO responsiveness to the intervention (Figure S1C). Regarding the Rb-treated group, the plasma TMAO levels of 6 subjects decreased, whereas TMAO increased in 11 subjects. Figure 1 illustrates the large inter-individual variability in plasma TMAO responses to Rb consumption. Based on post- versus pre-intervention TMAO delta values, subjects were then further assigned to the TMAO decrease (DEC, delta lower than zero) or increase (INC, delta equal or greater than zero) subgroups, respectively. The INC subgroup showed an increase in TMAO values of 134.2% after Rb consumption (from 2.95 ± 2.23 μmol/L at week 0 to 6.91 ± 4.68 at week 8), while the decrease in the DEC subgroup was 32.5% (from 5.47 ± 2.67 μmol/L at week 0 to 3.69 ± 1.39 at week 8). At week 0, no significant differences between the INC and DEC subgroups were observed for age, sex, anthropometric or metabolic variables (Table 1). Table 2 shows changes in metabolic and anthropometric and metabolic parameters from the beginning to the end of the intervention. No significant between-group differences in dietary intakes, anthropometric or metabolic variables in either follow-up or baseline data were found. Furthermore, within each subgroup, there were no significant differences in daily dietary intakes in pre- versus post-Rb (data not shown).

### 3.2. Gut Microbiota Composition According to the Plasma TMAO Response to the Rb Consumption

**Figure 2:**
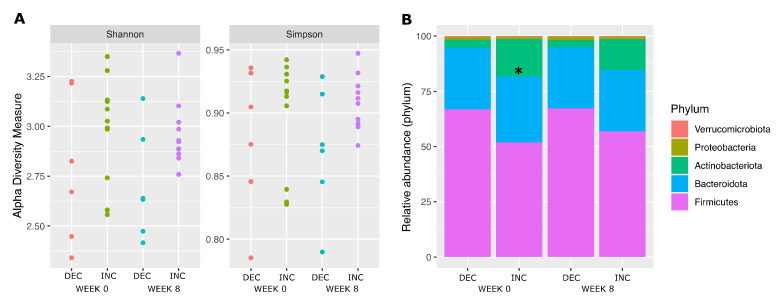
Composition of fecal microbiota according to TMAO subgroups. (**A**). α-diversity between TMAO subgroups according to Shannon (**left**) and Simpson’s reciprocal (**right**) indexes. Each participant is represented by a point. (**B**). Relative abundance of phyla among TMAO subgroups before and after Rb consumption. DEC and INC stand for TMAO decrease and TMAO increase subgroups, respectively. Asterisk stands for significant differences in INC versus DEC group at week 0.

**Figure 3:**
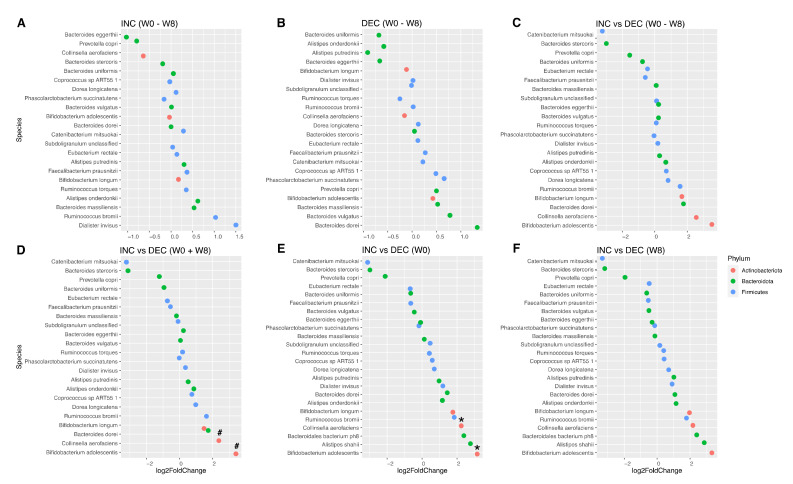
Differential abundance of fecal microbiota according to TMAO subgroups at the species level. Each bacterial species is represented by a point, and each color represents a different phylum. (**A**,**B**). Change in the abundance of bacterial species between weeks 0 and 8 in INC and DEC subgroups. (**C**). Differential change in the abundance of bacterial species between INC and DEC subgroups during the intervention. (**D**). Change in the abundance of bacterial species between CT and Rb groups, irrespective of the time. (**E**,**F**). Change in the abundance of bacterial species between CT and Rb groups at weeks 0 and 8, respectively. DEC and INC stand for TMAO decrease and increase subgroups, respectively. W0 and W8 stand for week 0 (baseline) and week 8 (end of the intervention). Asterisks and hashtags stand for significant differences between INC and DEC subgroups at nominal *p*-value < 0.05 and FDR-adjusted *p*-value < 0.05, respectively.

First, we sought to assess the potential impact of baseline microbiota on the heterogeneous levels of plasma TMAO observed throughout the intervention among all trial participants of the trial (Figure S1), but no significant differences were found between the Rb-treated group and the control group in either α-diversity, phylum and functional relative abundance (Figure S2) or in species differential abundance (Figure S3), at baseline or in response to the 8-week intervention. Gut microbiota alpha diversity was then compared between INC and DEC subgroups of the present study. Shannon’s diversity index and the Simpson’s reciprocal index were higher before and after the intervention in the INC subgroup when compared to the DEC subgroup (Figure 2A), although these differences failed to reach statistical significance. The relative abundance of Actinobacteriota was significantly higher in the INC group than in DEC at week 0 (*p* = 0.03), but this difference was no longer significant at week 8 (*p* = 0.07) (Figure 2B). There was no significant change in species abundance during the intervention in INC and DEC groups (Figure 3A,B, respectively), or a significant group-by-time interaction (Figure 3C). However, when comparing the INC and the DEC groups irrespective of the time, we found the abundance of two species significantly increased in the INC group (Figure 3D). These two species were *Bifidobacterium adolescentis* (FC = 3.5, FDR-*p* = 0.04) and *Collinsella aerofacines* (FC = 2.5, FDR-*p* = 0.038), both of which are part of the Actinobacteriota phylum, which had already been found to be significantly increased in the INC group at baseline. Similarly, when comparing INC and DEC groups by time, we found the same two species, *Bifidobacterium adolescentis* (FC = 3.9, *p* = 0.009) and *Collinsella aerofacines* (FC = 2.8, *p* = 0.02), significantly increased at week 0 (Figure 3E) but not at week 8 (Figure 3F).

### 3.3. Bacterial Species Associated with LBP and TMAO following Rb Consumption

Linear regression was used to investigate the association between changes between weeks 8 and 0 in bacterial species’ abundance and the plasma levels of TMAO and LBP. Linear regression analysis revealed significant associations between the abundance of several bacterial species and changes in both plasma TMAO and LBP levels. Overall, associations for a total of 24 fecal bacterial species with LBP and TMAO were established, among which 5 were common to LBP and TMAO, namely: *Granulicatella unclassified, Streptococcus_infantis, Flavonifractor_plautii, Anaerotruncus_colihominis and Bilophila wadsworthia* (Table 3 and Table 4).

**Figure 4:**
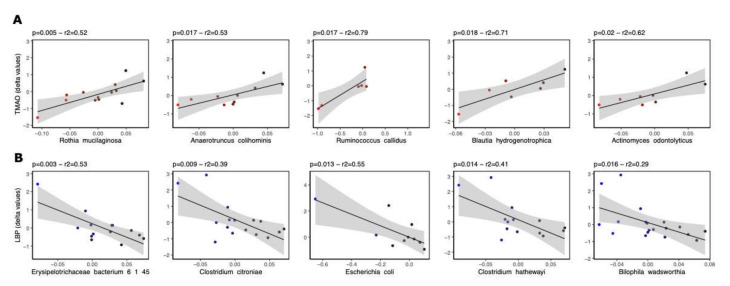
Association of fecal microbial abundance with plasma TMAO and LBP levels. The top five associations between fecal microbial abundance at the species level with TMAO and LBP plasma levels throughout the intervention (delta values) are shown in panels (**A**,**B**), respectively.

Using linear regression analysis, twelve bacterial species belonging to the following phyla were found to be associated with plasma TMAO changes: seven Firmicutes, three Actinobacteriota, one Bacteroidota and one Proteobacteria, while seventeen were found to associated with LBP: twelve Firmicutes, three Proteobacteria, one Bacteroidota and one Actinobacteriota. Out of the five common species, four were Firmicutes and one was a Proteobacteria (Figure 2). Bacterial species showing the strongest associations with plasma TMAO and LBP changes are shown in Figure 4A,B, respectively.

**Figure 5:**
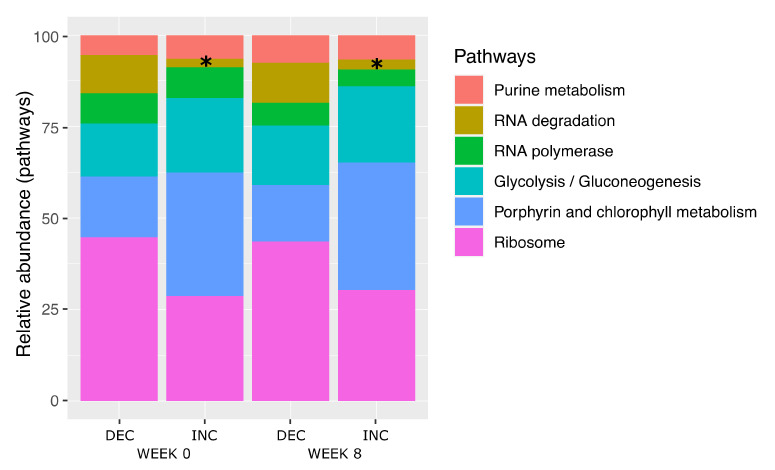
Relative abundance of the functional pathways of 12 fecal bacterial species associated with TMAO levels according to TMAO response subgroups. *p*-values were obtained using linear mixed models. Asterisks stand for significant differences in INC versus DEC groups either at week 0 or week 8.

There was an inverse direction of the associations between TMAO and LBP, i.e., all bacterial species’ abundance and plasma levels of TMAO were positively associated (except for *Collinsella aerofaciens*), but negatively associated with changes in plasma LBP (Table 3 and Table 4). Metagenomic analysis can also provide an opportunity to understand high-level functions. In total, we identified 63 KEGG pathways regarding the 12 bacterial species associated with TMAO levels, including 6 with a relative abundance greater than 10%, which were defined as dominant pathways (Figure 5). These pathways were related to purine metabolism, RNA degradation and polymerase, glycolysis/gluconeogenesis, porphyrin and chlorophyll metabolism and ribosome biogenesis. A significant group effect (INC vs. DEC) was noted, irrespective of time before or after Rb consumption, with regard to the RNA degradation pathway (*p* = 0.03).

## 4. Discussion

Interactions among diet, gut microbiota and plasma TMAO levels remain to be better characterized in humans, with somewhat contradictory findings. The purpose of this exploratory study was to point out potential associations between the plasma TMAO variability and gut microbiota in a context of Rb consumption. We have already reported that some individuals showed a strong increase in plasma TMAO after Rb consumption [4], which is contrary to the expected metabolic impact of these berries. In fact, since this increase in plasma TMAO levels is significantly different to plasma TMAO changes observed in the control group (Figure S1), it is plausible to consider this increase as potentially driven by the Rb consumption. Moreover, a significant part of the health benefits of Rb is attributed to its prebiotic potential, and the gut microbiota contribute to circulating concentrations of TMAO [1,14]. In view of that, the absence of a significant difference in baseline gut microbiota composition between participants from Rb-treated and control groups (Figure S2) led us to focus the study on Rb-consuming participants, clustering them into TMAO responders and non-responders to Rb consumption. In the present study, participants who showed a decrease in plasma TMAO levels during the intervention had a lower abundance of Actinobacteriota at week 0. Interestingly, in a porcine model of MetS, an association between gut microbial phyla and MetS parameters showed that Actinobacteriota was positively associated with MetS [15]. A cross-sectional study reported a higher relative abundance of Actinobacteriota in 12 elderly Chinese patients with MetS [16]. Similarly, a study of 172 Mexican children and adolescents showed that obese individuals had a higher relative abundance of Actinobacteriota than those of normal weight [17]. Thus, it appears that in some cases, the Actinobacteriota phylum is associated with metabolic disturbances. Interestingly, *Collinsella aerofaciens* and *Bifidobacterium adolescentis*, two species of the Actinobacteriota phylum identified herein as being differentially abundant between TMAO subgroups, have been previously found to be enriched in patients with symptomatic atherosclerosis [18,19], which is also closely related with increased plasma TMAO levels. These findings suggest that bacterial species from the Actinobacteriota phylum may potentially be involved in the unexpected increase in plasma TMAO levels following Rb consumption, but the underlying mechanisms are still yet to be elucidated.

In a recent study where TMAO was found to be associated with increased insulin resistance [20], the abundance of 13 genera was also significantly associated with plasma TMAO levels, including *Bilophila* and 2 genera from the *Ruminococcaceae* family [20]. In the present study, *Bilophila wadsworthia* and *Anaerotruncus colihominis* from the *Ruminococcaceae* family were positively associated with both plasma TMAO and LBP levels. The abundance of *Bi. wadsworthia*, which is a sulfite-reducing bacterium, was proved to be increased by the consumption of animal products [21]. Interestingly, the ability of the microbiota to synthesize TMA from phospholipids containing choline and L-carnitine depends on long-term dietary habits [2]. Therefore, the differences observed between subjects with increased or decreased plasma TMAO in pre- versus post-Rb consumption may be the result of pre-existing differences in gut microbial abundance as well as differences in consumption of meat products, although the latter differences were not observed in the present study.

The Western diet is characterized by a high caloric intake, high levels of animal proteins, fats and semi-essential nutrients containing quaternary amines, such as choline and carnitine. When the transport capacity of these quaternary amines through the small intestine is exceeded, they are exposed to colonic bacteria, which can convert them into TMA. However, a single-case study highlighted the small intestine as being the generation and absorption site of the phosphatidylcholines’ breakdown product TMA [1]. Remarkably, small intestinal bacterial overgrowth has been associated, on the one hand, with increased plasma TMAO levels in patients with heart failure [22], and on the other hand, with nonalcoholic steatohepatitis (NASH) [23]. Interestingly, small intestinal bacterial overgrowth subjects present an increased relative abundance of class Gammaproteobacteria in the duodenum [24], while patients with nonalcoholic fatty liver disease (NAFLD) present more abundant Gammaproteobacteria in stool samples [25]. In a clinical study in which choline intakes were manipulated in healthy women, the abundance of the microbial classes Erysipelotrichi and Gammaproteobacteria in stool samples was positively correlated with the development of fatty liver [26]. In the present study, *Erysipelotrichaceae bacterium 21 3* was positively associated with plasma TMAO levels. Interestingly, Erysipelotrichi abundance has been associated with high-fat intakes in animal models. Put concretely, a metagenomic analysis of humanized gnotobiotic mice that switched from chow to a Western diet indicated a bloom in Erysipelotrichi class members [27]. In this regard, switching mice with NASH from a low-fat diet to a Western diet resulted in changes in the gut microbial ecology towards a higher metabolism and conversion of choline to methylamines [28]. In parallel, a disturbance in choline metabolism, characterized by low circulating levels of plasma phosphatidylcholines and high urinary excretion of methylamines (dimethylamine, TMA and TMAO), has been observed [28]. Finally, LBP is part of a family of lipid transfer proteins and not only shuttles between bacterial LPS but also between phospholipids, including phosphatidylcholines [29]. In addition, a study highlighted that plasma LBP levels were significantly higher in children with NAFLD than in controls [30].

The major strength of the present study is the controlled nature of this nutritional intervention in humans as well as the deep phenotyping, including metabolomics and metagenomics. However, some limitations deserve to be acknowledged. First, the limited sample size upon which the present study is based constitutes a limitation. However, the heterogeneity observed in plasma TMAO levels during the intervention and the absence of an observable impact of baseline microbiota on them in the entire cohort led us to focus the study on the heterogeneity in the Rb-treated group. We recognize that focusing only on the Rb group may have introduced some bias and even limited the generalizability of the results. These are the limitations of the secondary studies that we have tried to address in order to produce valid results. In view of the results obtained, further and more targeted studies are warranted in the field to deepen the understanding on the inter-individual variability observed in gut-microbiota-derived factors such as TMAO. The interpretation of the present results is also limited by the absence of samples taken from the different segments of the intestine during the nutritional intervention, which would have undoubtedly enriched the discussion. In addition, the metabolomic analyses performed in the present study did not target phytochemical compounds, and thus did not allow us to relate TMAO results to potential metabotypes. Finally, the potential impact of sex on the clinical outcomes was not investigated due to the low number of subjects.

## 5. Conclusions

In summary, the results of this exploratory study suggest that the variability in the plasma TMAO response following Rb consumption we previously reported may potentially be the result of pre-existing differences in gut microbiota profiles between individuals. Changes in gut microbial ecology induced by Rb consumption resulted in large inter-individual variability, especially with regard to quaternary-amines-utilizing activity. Although associations cannot establish causality, results of the present study are in agreement with previous findings reported in animal and human studies. However, results presented herein must be interpreted with caution due to the absence of multiple-testing correction. Further research is still needed to clarify the relevance of the microbial species highlighted in the present study for the metabolism and conversion of quaternary amines into methylamines.

## Figures and Tables

**Table 1:** Clinical characteristics of study participants at week 0 according to TMAO subgroups.

Variable	N	DEC			N	INC			*p*-Value
Sex (men/women)	6	3/3			11	4/7			0.58
Age (years)	6	29.3	±	8.1	11	34.3	±	11.5	0.78
BMI (kg/m^2^)	6	30.5	±	3.7	11	30.0	±	5.9	0.89
Waist circumference (cm)	6	98.2	±	17.5	11	98.7	±	12.7	0.53
SBP (mmHg)	6	118.4	±	4.6	11	112.5	±	13.8	0.92
DBP (mmHg)	6	75.9	±	8.8	11	72.1	±	9.5	0.89
Total-C (mmol/L)	6	5.11	±	0.93	11	4.43	±	0.84	0.21
HDL-C (mmol/L)	6	1.23	±	0.59	11	1.34	±	0.36	0.88
LDL-C (mmol/L)	6	3.13	±	0.93	11	2.38	±	0.81	0.19
TG (mmol/L)	6	1.63	±	0.86	11	1.56	±	0.96	0.92
Fasting glucose (mmol/L)	5	5.54	±	0.83	11	4.96	±	0.43	0.21
Fasting insulin (pmol/L)	6	84.33	±	55.52	9	90.67	±	40.61	0.42
HbA1C (%)	6	5.05	±	0.33	11	5.05	±	0.31	0.77
LBP (μg/mL)	6	4.86	±	0.67	11	5.49	±	0.92	0.24
LPS (pg/mL)	6	76.02	±	19.87	11	87.09	±	19.63	0.33
CRP (mg/L)	6	2.30	±	2.07	10	1.81	±	1.10	0.57

Data are presented as raw means ± standard deviations. *p*-values are from Fisher’s exact test (sex) and general linear models adjusted for BMI, sex and age. Statistical significance was considered at *p* ≤ 0.05. BMI, body mass index. SBP, systolic blood pressure. DBP, diastolic blood pressure. Total-C, total cholesterol. HDL-C, high-density lipoprotein cholesterol. LDL-C, low-density lipoprotein cholesterol. TG, triglycerides. HbA1C, glycated hemoglobin. CRP, C-reactive protein. LBP, lipopolysaccharide -binding protein. LPS, lipopolysaccharide. DEC and INC stand for TMAO decrease and TMAO increase subgroups, respectively.

**Table 2:** Longitudinal changes between week 0 and week 8 in clinical characteristics of participants according to TMAO subgroups.

	DEC								INC								*p*-Values		
Variable	N	Week 0			N	Week 8			N	Week 0			N	Week 8			Group	Visit	Int
BMI (kg/m^2^)	6	30.5	±	3.7	6	30.4	±	3.9	11	30.0	±	5.9	11	30.2	±	6.1	0.56	0.76	0.51
Waist circ (cm)	6	98.2	±	17.5	6	98.7	±	12.7	11	98.7	±	12.7	11	99.1	±	13.2	0.66	0.78	0.48
SBP (mmHg)	6	118.4 ^a^	±	4.6	6	116.1 ^b^	±	6.9	11	112.5 ^a^	±	13.8	11	109.8 ^b^	±	13.7	0.89	**0.03**	0.84
DBP (mmHg)	6	75.9	±	8.8	6	72.6	±	6.1	11	72.1	±	9.5	11	70.1	±	11.3	0.36	0.08	0.48
Total-C (mmol/L)	6	5.11	±	0.93	6	4.99	±	0.95	11	4.43	±	0.84	11	4.20	±	0.69	0.31	0.21	0.69
HDL-C (mmol/L)	6	1.23	±	0.59	6	1.20	±	0.52	11	1.34	±	0.36	11	1.31	±	0.24	0.92	0.57	0.97
LDL-C (mmol/L)	6	3.13	±	0.93	6	3.08	±	0.90	11	2.38	±	0.81	11	2.33	±	0.66	0.45	0.68	0.99
TG (mmol/L)	6	1.63	±	0.86	6	1.54	±	0.93	11	1.56	±	0.96	11	1.21	±	0.49	0.30	0.30	0.54
Glucose (mmol/L)	5	5.54 ^a^	±	0.83	6	5.10 ^b^	±	0.72	11	4.96	±	0.43	11	4.95	±	0.55	0.80	**0.002**	0.15
Insulin (pmol/L)	6	84.33	±	55.52	5	71.60	±	26.99	9	90.67	±	40.61	10	103.70	±	64.42	0.63	0.49	0.79
HbA1C (%)	6	5.05	±	0.33	6	5.09	±	0.29	11	5.05	±	0.31	11	5.12	±	0.28	0.76	0.71	0.71
CRP (mg/L)	6	2.30	±	2.07	6	3.40	±	2.57	10	1.81	±	1.10	10	2.30	±	1.90	0.38	0.05	0.42
LBP (μg/mL)	6	4.86	±	0.67	6	5.53	±	1.47	11	5.49	±	0.92	11	5.27	±	0.89	0.44	0.40	0.11
LPS (pg/mL)	6	76.02	±	19.87	6	71.63	±	29.83	11	87.09	±	19.63	11	85.10	±	21.37	0.95	0.39	0.74

Data are presented as raw means ± standard deviations. *p*-values are from linear mixed models adjusted for BMI, sex, age and baseline levels. Statistical significance was considered at *p* ≤ 0.05 for the effects of group, visit and the interaction group by visit (Int). BMI, body mass index. SBP, systolic blood pressure. DBP, diastolic blood pressure. Total-C, total cholesterol. HDL-C, high-density lipoprotein cholesterol. LDL-C, low-density lipoprotein cholesterol. TG, triglycerides. HbA1C, glycated hemoglobin. CRP, C-reactive protein. LBP, lipopolysaccharide-binding protein. LPS, lipopolysaccharide. Glucose and insulin are fasting levels. Significant *p*-values are highlighted in bold. Letters stand for significant differences (^a^, ^b^ for visit differences). DEC and INC stand for TMAO decrease and TMAO increase subgroups, respectively.

**Table 3:** Association between fecal microbial abundance at the species level and plasma TMAO levels throughout the intervention.

Phylum	Class	Order	Family	Genus	Species	β-Value	*p*-Value	r^2^
Actinobacteriota	Actinobacteria	Actinomycetales	Actinomycetaceae	Actinomyces	Actinomyces odontolyticus	9.5	0.02	0.62
Actinobacteriota	Actinobacteria	Coriobacteriales	Coriobacteriaceae	Collinsella	Collinsella aerofaciens	−0.3	0.03	0.42
Actinobacteriota	Actinobacteria	Actinomycetales	Micrococcaceae	Rothia	Rothia mucilaginosa	9.5	0.01	0.52
Bacteroidota	Bacteroidia	Bacteroidales	Bacteroidaceae	Bacteroides	Bacteroides nordii	5.2	0.04	0.82
Firmicutes	Clostridia	Clostridiales	Ruminococcaceae	Anaerotruncus	Anaerotruncus colihominis	9.1	0.02	0.53
Firmicutes	Clostridia	Clostridiales	Lachnospiraceae	Blautia	Blautia hydrogenotrophica	20.0	0.02	0.71
Firmicutes	Erysipelotrichia	Erysipelotrichales	Erysipelotrichaceae	Erysipelotrichaceae	Erysipelotrichaceae bacterium 21 3	12.6	0.03	0.45
Firmicutes	Clostridia	Clostridiales	Clostridiales	Flavonifractor	Flavonifractor plautii	9.7	0.03	0.36
Firmicutes	Bacilli	Lactobacillales	Carnobacteriaceae	Granulicatella	Granulicatella unclassified	13.0	0.03	0.48
Firmicutes	Clostridia	Clostridiales	Ruminococcaceae	Ruminococcus	Ruminococcus callidus	1.8	0.02	0.79
Firmicutes	Bacilli	Lactobacillales	Streptococcaceae	Streptococcus	Streptococcus infantis	9.7	0.02	0.55
Proteobacteria	Deltaproteobacteria	Desulfovibrionales	Desulfovibrionaceae	Bilophila	Bilophila wadsworthia	17.9	0.02	0.37

Significant associations are from linear regression analysis *(p*-value < 0.05). β-value represents the direction of the association (positive or negative). r^2^ represents the determination coefficient of TMAO changes to the bacterial modulation; a larger value of r^2^ indicates a greater influence of TMAO changes on the bacterial modulation.

**Table 4:** Association between fecal microbial abundance at the species level and plasma LBP levels throughout the intervention.

Phylum	Class	Order	Family	Genus	Species	β-Value	*p*-Value	r^2^
Actinobacteriota	Actinobacteria	Coriobacteriales	Coriobacteriaceae	Adlercreutzia	Adlercreutzia equolifaciens	−11.0	0.02	0.25
Bacteroidota	Bacteroidia	Bacteroidales	Rikenellaceae	Alistipes	Alistipes finegoldii	−2.4	0.04	0.22
Firmicutes	Bacilli	Lactobacillales	Carnobacteriaceae	Granulicatella	Granulicatella unclassified	−14.4	0.04	0.32
Firmicutes	Bacilli	Lactobacillales	Streptococcaceae	Streptococcus	Streptococcus australis	−19.8	0.04	0.31
Firmicutes	Bacilli	Lactobacillales	Streptococcaceae	Streptococcus	Streptococcus infantis	−17.6	0.05	0.34
Firmicutes	Bacilli	Lactobacillales	Streptococcaceae	Streptococcus	Streptococcus mitis oralis pneumoniae	−17.6	0.05	0.31
Firmicutes	Clostridia	Clostridiales	Clostridiaceae	Clostridium	Clostridium citroniae	−15.0	0.01	0.39
Firmicutes	Clostridia	Clostridiales	Clostridiaceae	Clostridium	Clostridium hathewayi	−10.6	0.01	0.41
Firmicutes	Clostridia	Clostridiales	Clostridiales	Clostridiales	Clostridiales bacterium 1 7 47FAA	−14.9	0.03	0.41
Firmicutes	Clostridia	Clostridiales	Clostridiales	Flavonifractor	Flavonifractor plautii	−9.0	0.04	0.28
Firmicutes	Clostridia	Clostridiales	Lachnospiraceae	Lachnospiraceae	Lachnospiraceae bacterium 7 1 58FAA	−8.1	0.03	0.21
Firmicutes	Clostridia	Clostridiales	Ruminococcaceae	Anaerotruncus	Anaerotruncus colihominis	−16.4	0.05	0.32
Firmicutes	Erysipelotrichia	Erysipelotrichales	Erysipelotrichaceae	Erysipelotrichaceae	Erysipelotrichaceae bacterium 6 1 45	−17.5	0.00	0.53
Firmicutes	Erysipelotrichia	Erysipelotrichales	Erysipelotrichaceae	Holdemania	Holdemania filiformis	−14.1	0.04	0.27
Proteobacteria	Deltaproteobacteria	Desulfovibrionales	Desulfovibrionaceae	Bilophila	Bilophila wadsworthia	−14.0	0.02	0.29
Proteobacteria	Gammaproteobacteria	Enterobacteriales	Enterobacteriaceae	Escherichia	Escherichia coli	−4.4	0.01	0.55
Proteobacteria	Gammaproteobacteria	Enterobacteriales	Enterobacteriaceae	Escherichia	Escherichia unclassified	−7.8	0.03	0.53

Significant associations are from linear regression analysis *(p*-value < 0.05). β-value represents the direction of the association (positive or negative). r^2^ represents the determination coefficient of LBP changes to the bacterial modulation; a larger value of r^2^ indicates a greater influence of LBP changes on the bacterial modulation.

## Data Availability

Not applicable.

## Supplementary Materials

The following supporting information can be downloaded at: https://www.mdpi.com/article/10.3390/nu14081656/s1, Figure S1: Global and individual plasma changes in plasma TMAO levels after an 8-week raspberry consumption; Figure S2: Schematic overview of fecal microbiota composition and evolution according to control and raspberry-treated groups; Figure S3: Differential abundance of fecal microbiota according to CT and Rb groups at the species level. Each bacterial species is represented by a point and each color represent a different phylum.
