# Supplementary material for: Gut Microbial Signatures of Distinct Trimethylamine N-Oxide Response to Raspberry Consumption
Source: Nutrients. 2022 Apr 15;14(8):1656. doi: 10.3390/nu14081656 (PMC9027468; doi:10.3390/nu14081656)
Supplement: Supplementary file 1 [file nutrients-14-01656-s001.zip › nutrients-1642971-supplementary.pdf]

Supplementary material

Gut microbial signatures of distinct trimethylamine N-oxide response to raspberry consumption

Maximilien Franck[1,2,3], Juan de Toro-Martín[1,2,3], Thibault V. Varin[1,2,4], Véronique Garneau[1,2,3], Geneviève Pilon[1,2,4], Denis Roy[1], Patrick Couture[1,2,5], Charles Couillard[1,2,3], André Marette[1,2,4], Marie-Claude Vohl[1,2,3*]

1 Institute of Nutrition and Functional Foods (INAF), Université Laval, Québec, QC, G1V 0A6, Canada
2 Centre Nutrition, santé et société (NUTRISS), Université Laval, Québec, QC, G1V 0A6, Canada
3 School of Nutrition, Université Laval, Québec, QC G1V 0A6, Canada
4 Quebec Heart and Lung Institute (IUCPQ) Research Center, 2725 chemin Sainte-Foy, Québec, QC G1V 4G5, Canada
5 Endocrinology and Nephrology Unit, CHU de Quebec Research Center, Québec, QC G1V 4G2, Canada

* Correspondence: marie-claude.vohl@fsaa.ulaval.ca; Tel.: +1-418-656-2131 (ext. 404676)

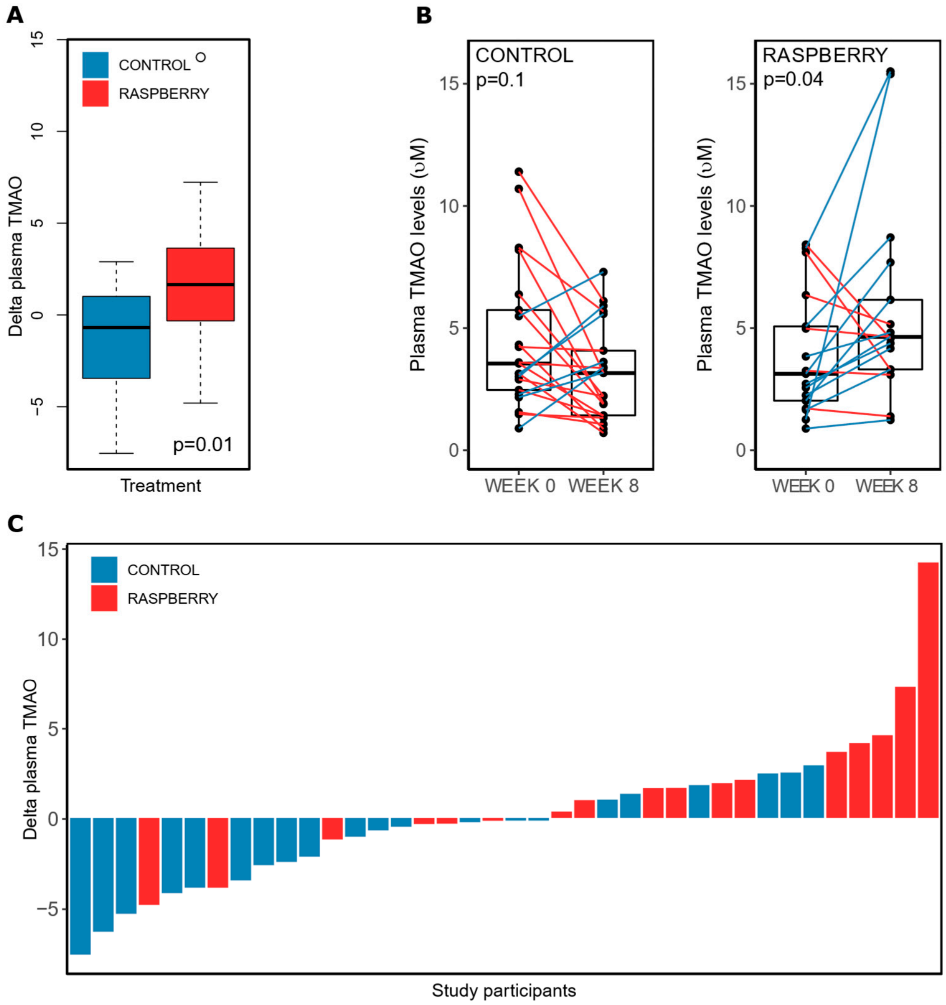

Figure S1. Global and individual plasma changes in plasma TMAO levels after an 8-week raspberry consumption. The y axis represents delta values in plasma TMAO in pre- versus post-intervention. **A.** Box and whisker plots show median, first, and third quartiles, and maximum and minimum values for the delta plasma TMAO values in the control (n=21) and raspberry-treated (n=17) groups. p stands for a p-value obtained from unpaired and two-tailed Student's *t* test. **B.** Plasma TMAO levels before (week 0) and after (week 8) the intervention in the control (n=21) and raspberry-treated (n=17) groups, respectively. Blue and red lines stand for increase or decrease in plasma TMAO levels, respectively, for each individual during the intervention. p stands for a p-value of the time effect obtained from linear mixed models. **C.** Each bar on the x axis represents

one participant from the control group (blue bars) and the raspberry-treated group (red bars).

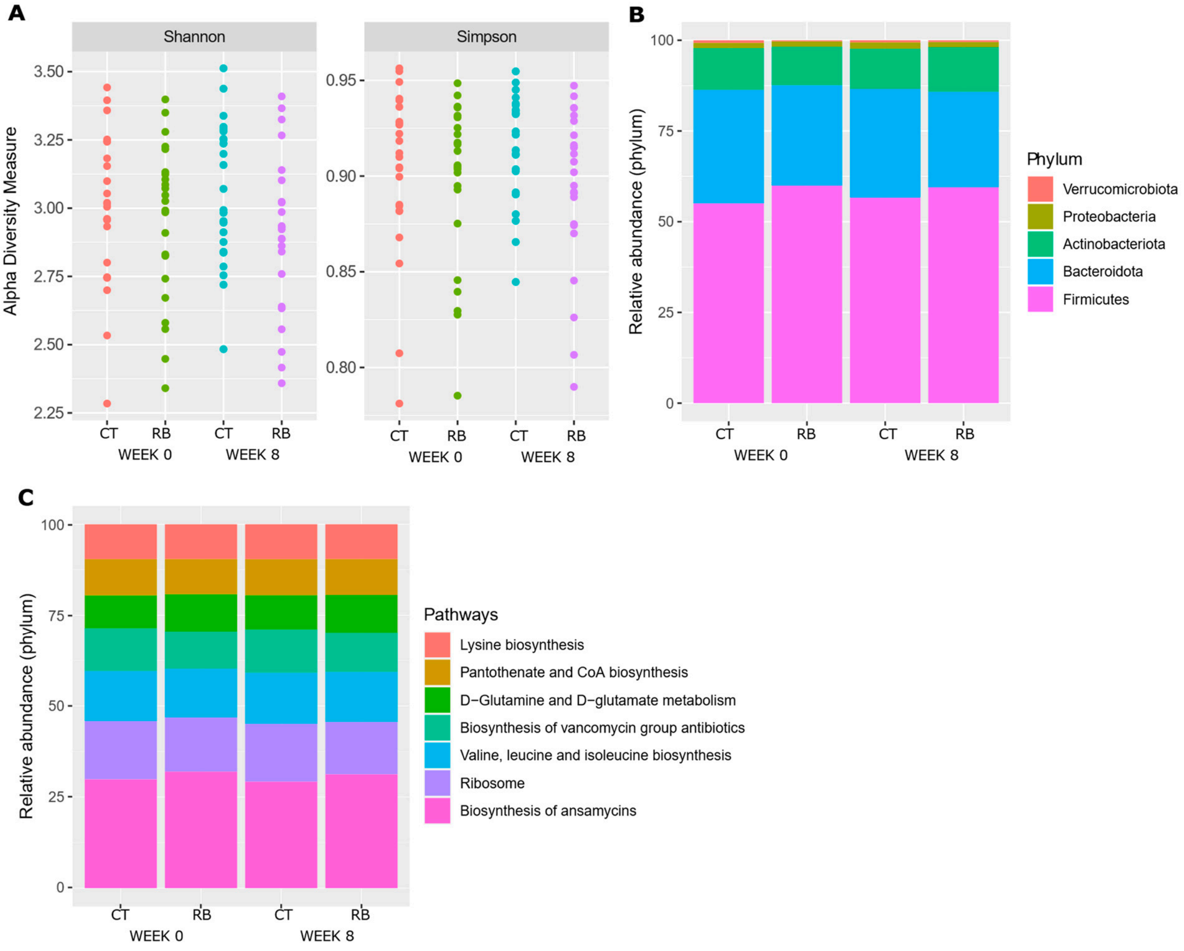

Figure S2. Schematic overview of fecal microbiota composition and evolution according to control and raspberry-treated groups. **A.** The differences in α-diversity between CT and RB groups according to Shannon (left) and Simpson's reciprocal (right) indexes are shown. Each point represents a participant. **B.** The relative abundance of bacterial phyla among CT and RB groups before and after the intervention is depicted. CT and RB stand for control and raspberry-treated groups, respectively. **C.** Relative abundance of the functional pathways showing a relative abundance greater than 10% and identified by using 23 bacterial species identified more than 3 times in at least 10% of the samples analyzed (n=34; 17 RB and 21 CT).

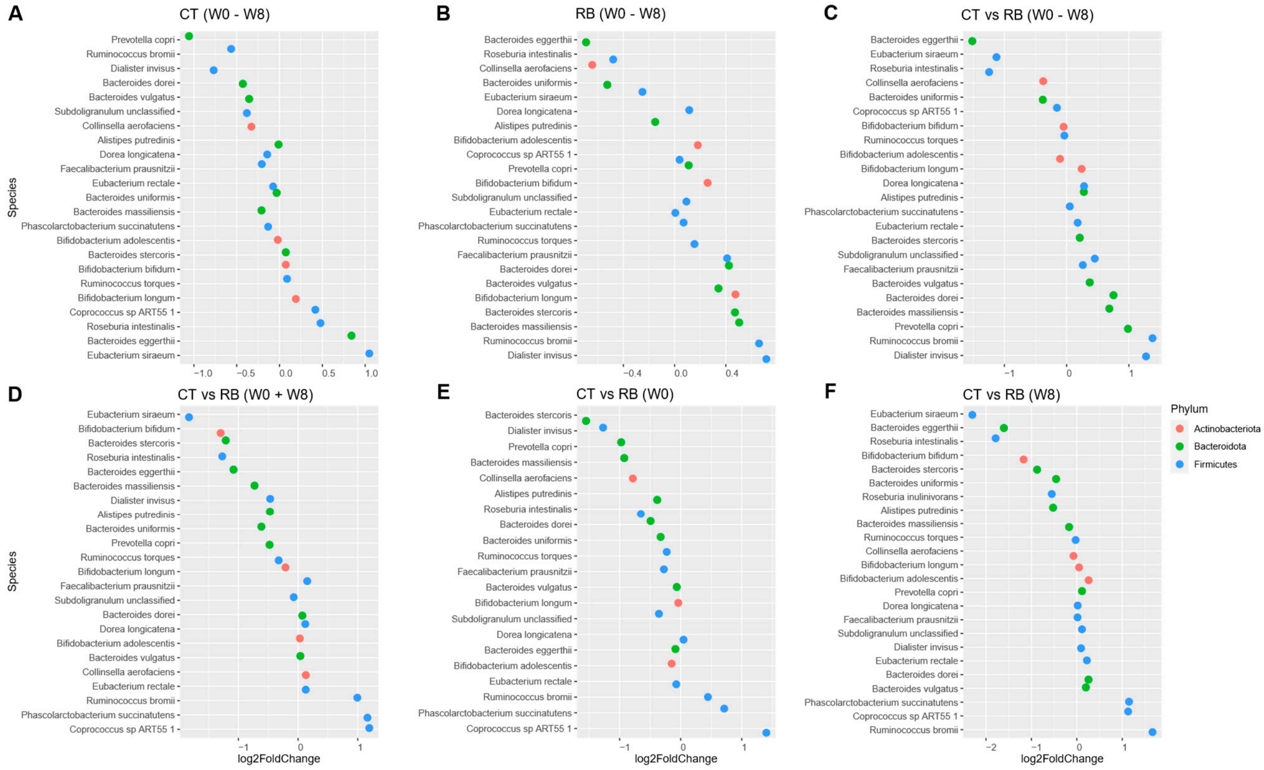

Figure S3. Differential abundance of fecal microbiota according to CT and RB groups at the species level. Each bacterial species is represented by a point and each color represent a different phylum. **A, B**. Changes in the abundance of bacterial species between weeks 0 and 8 in CT and RB groups. **C**. Differential change in the abundance of bacterial species between CT and RB groups during the intervention. **D**. Change in the abundance of bacterial species between CT and RB groups, irrespective of the time. **E, F**. Changes in the abundance of bacterial species between CT and RB groups at weeks 0 and 8, respectively. CT and RB stand for control and raspberry-treated groups,

respectively. W0 and W8 stand for week 0 (baseline) and week 8 (end of the intervention).
